# Editorial: Visual Timing Impairments in Developmental, Acquired, and Age-Related Neurological Conditions

**DOI:** 10.3389/fnhum.2020.640187

**Published:** 2021-01-14

**Authors:** Teri Lawton, John Stein, John Shelley-Tremblay

**Affiliations:** ^1^Department of Cognitive Neuroscience, Perception Dynamics Institute (PDI), Del Mar, CA, United States; ^2^Department of Physiology, Anatomy and Genetics, University of Oxford, Oxford, United Kingdom; ^3^Department of Psychology, University of South Alabama, Mobile, AL, United States

**Keywords:** visual timing, neurological pathways, developmental disorder, dyslexia, magno- and parvo-cellular systems, mild cognitive impairments, attention, feedback

Neural timing deficits have become increasingly implicated in neurological conditions, prompting the call for a special issue on this topic. The four papers selected for publication provide compelling new evidence that faulty timing in the interchange of information between magno and parvo-cellular processing streams could be a unifying theme that links Mild Cognitive Impairment (MCI, Chen et al.), Developmental Dyslexia (DD), and Autism Spectrum Disorder (ASD), but not Attention Deficit Hyperactive Disorder (ADHD, Brown et al.).

Knowledge of oscillatory entrainment and its fundamental role in cognitive and behavioral processing has increasingly been applied to research in the field of reading and developmental dyslexia. Growing evidence indicates that oscillatory entrainment to theta frequency spoken language in the auditory domain, along with cross-frequency theta-gamma coupling, support the phonological processing (i.e., the cognitive encoding of linguistic knowledge gathered from speech) which is required for reading. This theory is called the temporal sampling framework (TSF) and can extend to developmental dyslexia (Archer et al.), such that inadequate temporal sampling of speech-sounds in people with dyslexia results in poor theta oscillatory entrainment in the auditory domain, and thus a phonological processing deficit which hinders reading ability.

Similarly, Archer et al. suggest that inadequate theta oscillations in the visual domain might account for the many magno-dorsal processing, oculomotor control, and visual deficits seen in developmental dyslexia. They delineate the considerable evidence that indicates that visual deficits in dyslexia are associated with changes in the magnocellular pathway within the dorsal visual stream which result in difficulties encoding and processing the low-spatial, high-temporal frequency characteristics of reading. They hypothesize that this magno-dorsal deficit is one which affects neural synchrony within and between magnocellular dominated visual pathways. Archer et al. propose two possible models of a magno-dorsal visual TSF based on the coupling of theta and gamma oscillations: (1) A direct correlation whereby “bottom-up” magnocellular oscillatory entrainment of the visual domain by magnocellular populations phase locks to theta frequency eye movements during reading or (2) an inverse correlation by which attending to text triggers “top-down” low gamma signals from higher-order visual processing areas that organize magnocellular populations to synchronize to the theta frequency, and this drives the timing of eye movements and captures letter images at a higher frequency.

Chen et al. found that older adults with mild cognitive impairments (MCI) have slower response times when doing direction discrimination relative to congruent and incongruent flankers. They propose that these slower response times result from prestimulus alpha oscillations, conveyed by magnocellular neurons, not being lower for fast patterns, as is found for normal healthy controls. This study also suggests that deficits in focusing attention are due in part to the temporal inaccuracy of magnocellular neurons. Not only do magnocellular neurons provide crucial input to attention networks, but they also provide input for gauging emotional salience, which acts similarly to attention. Mu and Crewther show that negative facial emotional information (fearful v. happy or neutral faces) is conveyed rapidly by magnocellular neurons to V1 and thence to the amygdala. This activates the pulvinar that projects onto the Thalamic Reticular Nucleus to decrease the activity of magnocellular neurons in the thalamus (McAlonan et al., 2008) thus acting as an attention gate keeper. Mu and Crewther propose that this arrangement mediates a contest between negative feedback and response gain modulation.

These papers show that high level cognitive tasks, like reading, face perception, and direction discrimination relative to congruent and incongruent flankers, require both response gain modulation of attention networks by magnocellular neurons and also feedback by coupled theta/gamma and alpha/gamma oscillations, as proposed previously by Lawton (2016) and Lawton and Shelley-Tremblay (2017). These studies imply that improving inefficient magnocellular function using tasks designed to optimally activate magnocellular neurons relative to linked parvocellular neurons, should improve not only visual motion but also attention and executive control networks. This can be achieved by slowly increasing the speed of a moving target from theta (6.5 Hz) to alpha frequencies (13.3 Hz), which increases both the speed and sensitivity of left-right movement discrimination relative to stationary backgrounds (Lawton, 2016; Lawton and Shelley-Tremblay, 2017). The studies in this special issue show that abnormalities with neural oscillations are linked to inefficient temporal processing, which may underlie a wide range of neurodevelopmental disorders. These studies contribute to a wider literature suggesting that this deficiency can demonstrate neuroplasticity and is amenable to improvement subsequent to proper exercises that optimally activate magnocellular neurons relative to linked parvocellular neurons.

## Author Contributions

TL wrote the initial manuscript. JS-T and JS edited the manuscript. JS-T submitted the manuscript. All authors contributed to the article and approved the submitted version.

## Conflict of Interest

The authors declare that the research was conducted in the absence of any commercial or financial relationships that could be construed as a potential conflict of interest.
